# Application of robot navigation system for insertion of femoral neck system in the treatment of femoral neck fracture

**DOI:** 10.1186/s12891-024-07172-z

**Published:** 2024-01-10

**Authors:** Gang Liu, Chengzhi Yang, Renchong Wang, Jingli Tang, Hao Wu, Lu Wei, Juzheng Hu

**Affiliations:** Department of Trauma Center, Liuzhou Worker’s Hospital, The Fourth Affiliated Hospital of Guangxi Medical University, Guangxi, 545005 China

**Keywords:** Orthopaedic surgery robot navigation system, Orthopaedic robot, Femoral neck fractures, Femoral neck system, Three-dimensional navigation

## Abstract

**Purpose:**

To evaluate the short-term clinical efficacy and advantages of surgery robot positioning system for insertion of Femoral Neck System (FNS) in the treatment of femoral neck fractures.

**Methods:**

The clinical data of 52 patients with Femoral neck fracture (FNF) who had been treated with FNS between June 2020 and September 2021 were retrospectively analyzed. Among them, 26 patients were treated with traditional FNS (control group), while 26 additional patients were treated with FNS assisted by an orthopaedic robot positioning system (study group). The operation duration, frequency of key-guide needle placement, intraoperative blood loss, incision length, fracture healing rate, fracture healing time, and the Harris scores at the last follow-up were calculated and compared between the 2 groups.

**Results:**

The study group had shorter operation duration, fewer numbers of placing the key-guide needle, less intraoperative blood loss, and smaller surgical incisions than the control group (all, *P* < 0.05). There was no significant difference in the rate of fracture healing rate between the 2 groups (*P* = 0.47), while the fracture healing duration of the study group was shorter than that of the control group (*P* = 0.03). At the last follow-up, compared with the control group, the Harris score and the number of excellent and good ratings were significantly higher in the study group (all, *P* < 0.05).

**Conclusions:**

Using orthopaedic surgery robot positioning system-assisted FNS in the treatment of FNFs can effectively improve the efficiency of surgery, shorten operation time, and reduce the number of placing the key-guide needle, intraoperative blood loss, and operative trauma. Simultaneously, it shortens the duration of fracture healing and improves the recovery of hip function.

## Introduction

Femoral neck fractures (FNFs) are the most common type of hip fracture, globally, the incidence of FNFs is increasing yearly as the world population is ageing [1]. The surgical treatment for FNFs can be either arthroplasty or internal fixation, depending on fracture type, bone quality, and patient’s age [2]. At present, the most commonly used implants are multiple cannulated screws (CS), dynamic hip screws (DHS), and femoral neck system (FNS), furthermore, the recently developed FNS, consists of the theoretical mechanical advantages of combining compression and anti-rotation qualities, showing promising biomechanical and clinical results compared to other implants for the treatment of FNFs [3–5]. Unfortunately, the incidence of postoperative complications remains high [3, 4, 6], such as fracture nonunion, cut out, and osteonecrosis of the femoral head.

Some studies have confirmed that exact internal fixation placement is closely related to fracture stability and fracture healing, and reduce the risk of fracture nonunion [4, 7]. However, the optimum internal fixation placement brings great difficulty to the operation. The traditional method of internal fixation placement for FNFs is usually operated by surgeons with a wealth of experience in manual positioning under fluoroscopy. In recent years, with the development of artificial intelligence, imageology, and robotics, the orthopaedic surgery robot positioning system technique has emerged as a new technology and applied to assist the operation of minimally invasive and precise internal fixation [8]. It has been demonstrated that an orthopaedic robot has been applied in the treatment of FNFs with cannulated screw internal fixation, additionally, the robot can improve the accuracy of the placement direction of the guide needle and the effect of surgical treatment, shorten the operation duration, reduce the surgical trauma and the injury caused by X-ray, compared with the conventional non-navigated technique [9–11]. With those significant advantages, the orthopaedic robot has shown great value in clinical application and has attracted growing attention. However, clinical data on the Robot-assisted FNS internal fixation of FNFs has not been reported so far.

The purposes of this retrospective study were: (i) to compare the short-term clinical efficacy of FNS assisted by the orthopaedic surgery robot positioning system and traditional FNS in the treatment of FNFs; (ii) to investigate the advantages and limitations of a robot-assisted positioning system for insertion of FNS in the treatment of FNFs.

## Materials and methods

### Inclusion and exclusion criteria

The inclusion criteria included: (i) patients aged between 18 and 65 years old and diagnosed with unilateral closed FNFs by X-ray or CT; (ii) The time from injury to surgery was less than 3 days; (iii) patients treated with FNS by using the robot navigation system or in the conventional method; (iv) good blade position and reduction quality were obtained.

The exclusion criteria included: (i)unable to tolerate surgical treatment due to comorbidities, such as severe liver, kidney, or cardiovascular disease; (ii) the patients received open reduction and internal fixation; (iii) pathological fracture;(iv) patients had a history of moderate to severe hip arthritis or osteonecrosis of the femoral head. (v) the postoperative follow-up period was less than 6 months.

### General clinical data

The clinical data of 52 patients with FNF who had been treated with FNS between June 2020 and September 2021 were retrospectively analyzed. According to surgical procedures, patients were randomly divided into 2 groups, each group consisting of 26 patients. All patients underwent preoperative CT and anteroposterior and lateral, and fracture type was recorded using the Garden classification [12]. Fractures were deemed stable if classified as types I or II and unstable for types III or IV. The study was approved by the ethics committee of our hospital, all patients signed informed consent for surgery.

### Operative procedure (traditional group)

In the traditional group, the FNS was inserted with the use of a standard C-arm fluoroscope in conventional two-dimensional mode.

After general or epidural anaesthesia, the patients were placed in an orthopaedic traction bed, with proper traction for closed reduction of the fracture. Quality of reduction was evaluated by radiographic measurements performed on initial postoperative anterior-posterior and lateral radiographs, measurement of the quantitative indicators proposed by Haidukewych et al. [13], as follows: excellent reduction (displacement after reduction <2 mm and deformity angle <5°), fair reduction (displacement ranging from 6 to 10 mm and deformity angle ranging from 11°– 20°), poor reduction (displacement > 10 mm and deformity angle >20°). A 2.0-mm Kirschner wire was placed to sustain fracture reduction. Then a key guide was inserted along the femoral neck using the 130° guide, the needle was located as close to the centre of the femoral neck as possible and kept 5 mm of the cartilage of the femoral head. If there was a deviation in the location of the guide needle, it was pulled out for repositioning. After a satisfactory location was achieved, the bone marrow channel was drilled along the guide needle and the depth was measured to determine the appropriate size of internal fixation. The implant and the anti-rotation screw were placed through the insertion handle using the same entry, subsequently, the distal locking screw was inserted using an additional entry. C-arm fluoroscopy was performed again to confirm the implant position, if proper, the subcutaneous tissues and the skin were sutured.

### Operative procedure (tirobot group)

In the TiRobot group, the FNS was inserted with the use of the third generation of the TIANJI orthopaedic robot, TiRobot (TINAVI Medical Technologies, Beijing, China), and the C-arm fluoroscope in three-dimensional mode. General Procedure was referred to the method described before [14, 15].

**Fig. 1:**
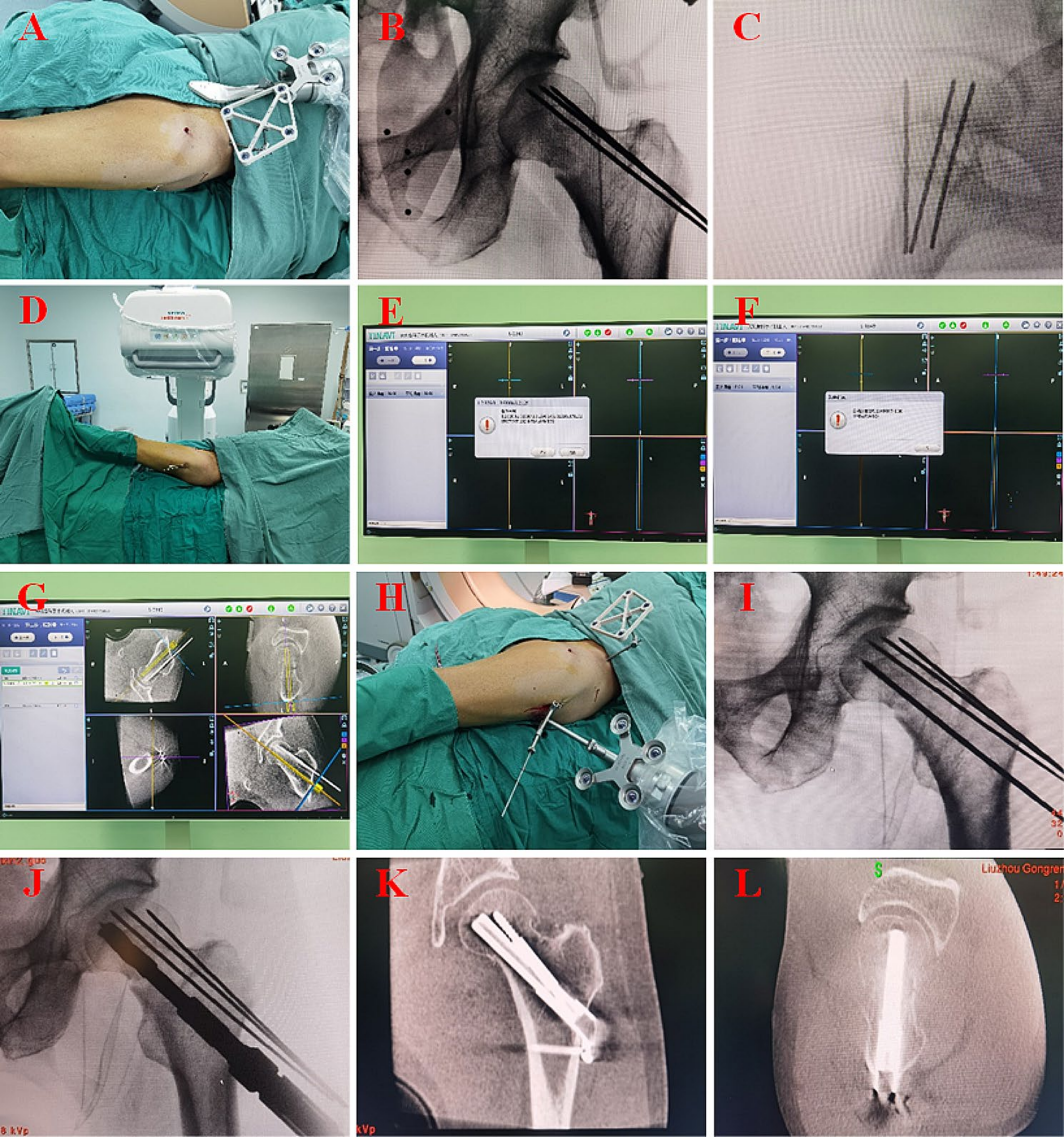
The operative procedure in TiRobot group. **(A)** Preparation of the Robot, **(B, C and D)** Navigation image acquisition, **(E and F)** Image registration, **(G)** Surgical path planning, **(H)** Mechanical arm operation and guide pin placement, **(I)** Guide pin verification, **(J)** Reamed along the guide pin, **(K and L)** C-arm 3D imaging confirmed that fracture reduction and implant location were almost satisfactory

**Fig. 2:**
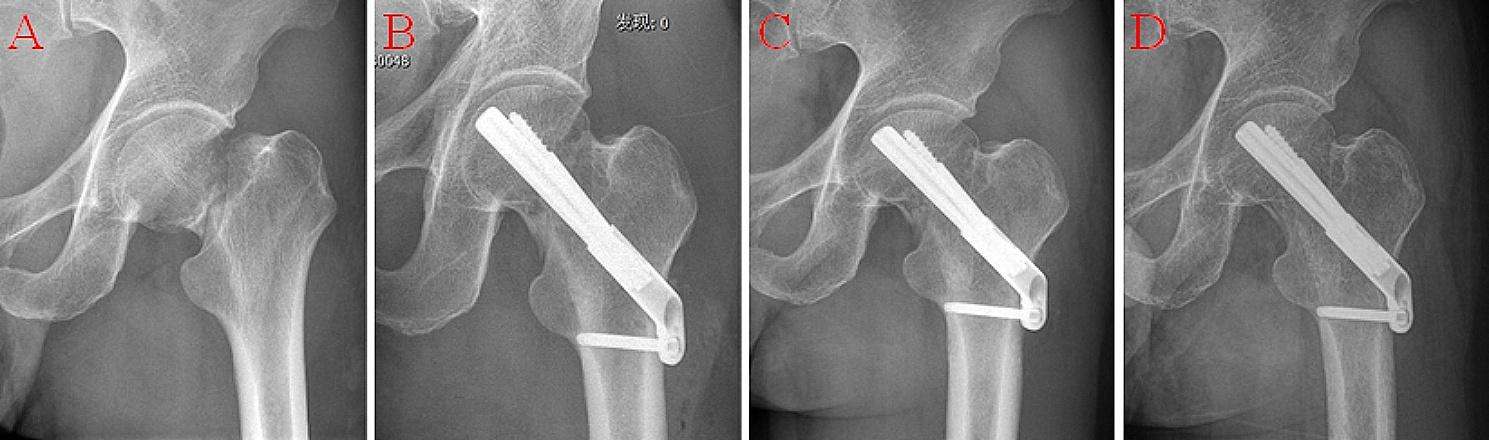
Radiograph of the femoral neck after the surgery: **(A)** preoperative, **(B)** postoperative, **(C)** 3 months postoperative, **(D)** 6 months postoperative

After achieving adequate anaesthesia and closed reduction of the fracture, an optical tracer was placed in the anterior superior iliac spine on the affected side and the robot tracer was assembled after the robot arm was fixed with a sterile protective sleeve (Fig. 1. A) and anteroposterior and lateral radiographs of the hip were obtained to ensure the robot tracer within fields of view imaged (Fig. 1. B, C). The C-arm in three-dimensional mode is employed to acquire intraoperative fluoroscopy images by rotating 180° around hip joints (Fig. 1. D) and transmitting them to the navigation system for three-dimensional imaging and registration calculation (Fig. 1. E, F). The location of the key-guide needle was then planned on the reconstructed three planes (anteroposterior, lateral, and coronal plane) (Fig. 1. G). The optimal position for FNS was at the centre of the femoral neck (lateral view), centre or inferior of the femoral neck (anteroposterior view), and 5 mm below the cartilage of the femoral head. After the surgeon had defined satisfactory implant positions and the robot had simulated the operation posture of the mechanical arm, the control software of the robot regulated the movement of the mechanical arm with the guide sleeve along the planned trajectory to the target location (Fig. 1. H, I). After key-guide needle placement, the implants were placed in the same manner as in the traditional technique (Fig. 1. J, K, and L). Typical Case: There was a 58-year-old male patient in the study group with a fracture of the right femoral neck. He was treated with robot-assisted FNS internal fixation (Fig. 2. Radiograph of the femoral neck after the surgery).

### Postoperative management and observation indicators

Both groups of patients underwent routine anti-infective and anti-thrombosis treatment, and reduction and internal fixation were determined by radiography and computed tomography on the day following surgery. All patients were regularly followed up once a month after surgery, imaging re-examination was conducted to evaluate fracture healing, and the rehabilitation program was adjusted according to the review results.

The general surgical conditions, including operative duration (taken from the end of fracture reduction, until the wound was sutured), frequency of key-guide needle drilling, intraoperative blood loss, and incision length were compared between the two groups. Additionally, the clinical efficacy of the two groups of patients was compared, including the fracture healing rate, fracture healing time, the Harris scores, and the number of excellent ratings of hip function at the last follow-up. The Harris score system [16] was applied to evaluate hip function, which includes four aspects: pain, function, deformity, and range of motion. Clinical efficacy was graded as follows: excellent (90–100), good (80–89), fair (70–79), and poor (< 70).

### Statistical analysis

Statistical analysis was performed using SPSS (IBM SPSS 23.0, SPSS Inc). Continuous variables were presented as means and standard deviation (SD) and compared using *a t*-test when the data were normally distributed, whereas characteristics with non-normal distributions were presented as median (Quartiles), and the *Mann*-*Whitney U* test was performed. Categorical variables are presented as frequencies and percentages, and a chi-squared test or Fisher exact test was performed to compare the proportions of categorical variables. *p*-values less than 0.05 (*P* < 0.05) were considered significant.

## Results

### Characteristics of patients

In the study group, 26 patients (9 males and 17 females) were aged from 35 to 65 years, with an average age of 50.08 years. In the control group, 26 patients (11 males and 15 females) were aged from 31 to 64 years, with an average age of 51.92 years. Patient characteristics are presented in Table 1(Patient characteristics of the two groups). There were no significant statistical differences in gender, age, cause of injury, injury side, and fracture type between the two groups, and they were comparable (*P* > 0.05).

### Intraoperative results

All patients achieved satisfactory postoperative reduction and a good blade position. The study group had shorter operation duration, fewer numbers of placing the key-guide needle, less intraoperative blood loss, and smaller surgical incisions than the control group (all, *P* < 0.05). Details are shown in Table 2(Comparison of the intraoperative results between the two groups).

### Clinical efficacy

All patients were followed up for 6 to 12 months. There was no infection, no loosening of internal fixation, no fracture displacement or osteonecrosis of the femoral head, or other complications in the study group during the follow-up period, and the fracture healing rate was 100% (26/26). While two nonunions happened in the control group which did not affect the quality of life and follow-up is ongoing, the fracture healing rate was 92.3% (24/26). The difference was not statistically significant between the two groups (*P* = 0.47). However, patients in the study group showed shorter fracture healing time than in the control group (*P* = 0.03, Table 3). The Harris score was significantly higher in the study group compared to the control group (*P* = 0.03). In the study group, the clinical curative effect was excellent in 17 cases, and good in 8 cases, the excellent and good rate was 96.2% (25/26). In the control group, the clinical curative effect was excellent in 9 cases, good in 9 cases, the excellent and good rate was 69.2% (18/26). There was a significant difference between the two groups (*P* = 0.03, Table 3. Comparison of the clinical efficacy between the two groups).

**Table 1:** Patient characteristics of the two groups

Patient characteristics	Control group (*n* = 26)	Study group (*n* = 26)
Gender (male/female)	11/15	9/17
Age (years), mean (SD)	50.08(8.41)	51.92(6.41)
Causes of injury		
Falls	8	6
Sports	3	7
Traffic accidents	15	13
Injury side (n, Left/Right)	16/10	12/14
Garden classification (n)		
I	3	2
II	7	6
III	13	15
IV	3	3

**Table 2:** Comparison of the intraoperative results between the two groups

Characteristics	Control group (*n* = 26)	Study group (*n* = 26)	t/Z/χ value	*P* value
Time to surgery (hours), mean (SD)	19.8(17.0)	24.4(18.2)	0.93	0.36
Operation duration (minutes), mean (SD)	55.5(7.48)	49.5(6.45)	3.1	0.03
Frequency of key-guide needle drilling (n), median (Q1, Q3)	4(3, 4)	1(1, 2)	-5.65	0.00
Intraoperative blood loss (mL), mean (SD)	59.5(13.48)	41.46(15.45)	4.48	0.00
Incision length (cm), mean (SD)	5.06(0.75)	3.05(0.28)	12.91	0.00
Optimal blade position, n (%)	23(88.5)	26(100)	3.18	0.24

**Table 3:** Comparison of the clinical efficacy between the two groups

	Control group (*n* = 26)	Study group (*n* = 26)	χ/t value	*P* value
The fracture healed cases, n (%)	24(92.3)	26(100)	0.52	0.47
Fracture healing duration (months), mean (SD)	4.38(0.88)	3.85(0.78)	2.25	0.03
Harris score, mean (SD)	81.42(6.61)	85.27(6.56)	2.11	0.04
Excellent, n (%)	9(34.6)	17(65.4)		
Good, n (%)	9(34.6)	8(30.8)		
Average, n (%)	6(23.1)	1(3.8)		
Poor, n (%)	2(7.7)	0(0)		
Excellent and good, n (%)	18(69.2)	25(96.2)	4.84	0.03

## Discussion

The most important finding of the study was that FNS assisted by the orthopaedic surgery robot positioning system is an ideal method for the treatment of FNFs. This technique can effectively improve the efficiency of surgery, shorten operation time, and reduce the number of placing the key-guide needle, intraoperative blood loss, and operative trauma. Simultaneously, it shortens the duration for fracture healing and improves the recovery of hip function and therefore prognosis.

For the FNF in younger adults, anatomical reduction and reliable internal fixation are generally preferred [1]. There are various methods of internal fixation, including multiple CS, DHS, Hansson Pins, and FNS. Among these, the FNS provides a stronger fixation and improves axial and rotational stability as a result of the screw-plate construct and the combination of blade and anti-rotation screw. Some studies [17, 18] have shown the biomechanical stability superiority of FNS over the CS, DHS, and Hansson Pins. The imprecise location and direction of an implant are closely related to bad clinical efficacy, such as loosening of internal fixation, fracture displacement, and osteonecrosis of the femoral head. Therefore, the proper location and depth of internal fixation are the key to the success of the operation. However, traditional freehand FNS placement is often influenced by the experience of surgeons. Due to human vision errors and unstable operation, it is difficult to ensure that the location and angle of implants are standard and the malposition rate is very high, which directly leads to postoperative complications and influences the outcome of the surgery. With the continuous development of computers, imageology, and robotics, orthopaedic surgery robot positioning system technology has been accepted by more and more surgeons and is widely used in surgical operations. By integrating X-ray or three-dimensional CT image imaging data, the robot can assist surgeons performing a more reasonable treatment, improve accuracy, and avoid human operator error [19, 20]. Femoral neck fracture internal fixation using three-dimensional navigation robotic-assisted FNS placement is undertaken in this study.

In this study, the results showed that the study group had shorter operation duration, less intraoperative blood loss, and smaller surgical incisions than the control group (all, *P* < 0.05). The operation duration included the non-invasive robot path planning time and the invasive fixation time in this study, thereby, the duration of the operation from the moment of inserting the key-guide needle until the subcutaneous tissues and the skin were sutured was significantly less than in the control group. With the operation experience of the robot increased, freehand screw insert might be more time-consuming than the programmed operation, such as equipment debugging, image acquisition, and path planning. In the traditional group, the guide needle was only roughly located and the incision sometimes should be appropriately extended in case the implants were blocked by skin and soft tissue. Different from the traditional operation, the skin was incised after the guide needle had been inserted, and the subsequent operations were performed without the need for extending the incision to expose the proximal femur. The mean length of the incision was only 3 cm, which was more minimally invasive in the study group. By contrast, smaller incisions and shorter surgery time had the advantages of less intraoperative blood loss in the study group, and function recovered more rapidly after surgery, especially in the elderly populations.

Overmuch drill attempts might weaken the cortical and cancellous bone resulting in subtrochanteric fractures [21, 22]. In the present study, the robot provided accurate navigation capabilities and a stable mechanical arm, avoiding repeated drilling resulting from the inevitable hand instability, and the frequency of key-guide needle drilling in the study group was significantly lower than that in the control group. This may represent the major advantage of this technique. The fewer number of placing the key-guide needle resulted in less trauma and reduced intraoperative blood loss. The extra fluoroscopy exposure could be reduced theoretically because of that the guide needles do not need to be adjusted repeatedly. However, a three-dimensional computer-assisted navigation technique was used in the present study and the number of fluoroscopic shots increased significantly compared to the two-dimensional technique. The complete and accurate three-dimensional information could provide a better service to high-precision orthopaedic surgery and deliver a more accurate localization of the guide needle, as He et al. [23] reported that 16.67% of patients with screws exiting the posterior cortex in the robot group, which using 2D planar navigation robotic-assisted cannulated screw placement. Moreover, a single scan of the C-arm in three-dimensional mode was needed only for the robot-assisted navigation, and further rotation of the C-arm was not necessary, it was beneficial for the aseptic conditions.

A variety of factors is known to influence the outcome of patients with FNFs. such as surgical method, the timing of surgery, fracture type, blade position and reduction quality. FNS assisted by the orthopaedic robot positioning system is a new surgical method for the treatment of FNFs. In the evaluation of the clinical efficacy after the operation, patients with robot-assisted treatment also showed explicit advantages. The results show that the new method can effectively shorten the operation time and incision length, reduce the amount of blood loss and improve the accurate location of the guide needle. These factors improved the safety and accuracy of surgery and promoted the postoperative recovery of the human body. Compared with the control group, the Harris score and the number of patients with an excellent or good rating after the operation were significantly higher in the study group (all, *P* < 0.05). During the follow-up period, the present study revealed that Robot-assisted surgery did not increase the fracture healing rate(*P*>0.05), however, patients in the study group showed shorter fracture healing time than in the control group (*P* < 0.05), this might benefit from fewer numbers of placing the key-guide needle. Overmuch drill attempts might weaken the cancellous bone and the local blood supply was destroyed and more time might be required for blood supply recovery. There was no infection, no loosening of internal fixation, no cut-out, no fracture displacement or osteonecrosis of the femoral head, or other complications in the study group during the follow-up period. Two nonunions happened in the control group which did not affect the quality of life and follow-up is ongoing. Many predisposing factors are associated with failures in FNFs, including suboptimal fracture reduction, poor implant positioning, more complex fracture types, older age, female sex and less experienced surgeons [24–26]. Of these, the blade position might be improved with the help of navigation. Suboptimal positioning might precipitate an elevation in stress concentrations on both the screw and plate, consequently predisposing the patient to a spectrum of potential complications [27]. However, a longer follow-up will be required to observe long-term complications and larger sample data are required to analyze the clinical efficacy.

The orthopaedic surgery robot positioning system assisted FNS in the treatment of FNFs possesses a wide range of applications and has the following advantages over traditional surgery. (i) Programmed surgical procedures. Robot-assisted orthopaedic surgery is a process of eye-brain-hand coordination. The procedure only required image acquisition and recognition, subsequently, the robot hosts provided prompts to complete the surgical design, registration, tool tracking, and navigation, and the implants can be precisely inserted according to standard procedures. (ii) Precise localization. After surgical design and registration, the robot can accurately and stably place the guide sleeve on the surgical site through the mechanical arm according to the kinematics parameters. (iii) Correction function. If the entry point and direction of the virtual guide wire deviate from the plan, the surgeon can fine-tune the path with the robot. (iv) Minimally invasive. Different from the traditional operation, the skin was incised after the guide needle had been inserted, and the subsequent operations were performed without the need for extending the incision to expose the proximal femur. The intraoperative blood loss and the severity of surgical trauma were reduced. (v) Reduced radiation load. Numerous studies [9, 11, 23, 28, 29] have confirmed that advantage, regrettably, no such advantage was detected in this study because of our use of the C-arm fluoroscope in three-dimensional mode. However, for the surgeon, the radiation dose was reduced compared to the conventional method, as the automated rotating scanning procedure was standardized and the navigation system allowed them to leave the operation room during 3D Scan. While there was an increase in radiation for the patient. The more idealized technique is to combine intraoperative X-ray with preoperative CT, achieving two or three-dimensional registration, which retains 3-dimensional spatial information and improves the accuracy of screw placement meanwhile reducing the intraoperative fluoroscopy duration. Looking forward to this technique.

However, there are still some deficiencies in the navigation robot system. (i) The navigation robot system relies on the experience of surgeons to implement the surgical path planning, missing teaching of operating technique principles, and there may be an error caused by subjective judgment. (ii)The guide needle may slide relative to the bone surface and a slight deviation from the original planning path may arise, when the guide needle passes through the lateral wall of the femur, because of the stiff cortical bone. (iii) An optical tracer was placed in the anterior superior iliac spine on the affected side for the image collection, and this extra-invasive manipulation may not be beneficial for these patients. In theory, the optical tracer should be tethered to the femur, however, this might hamper the follow-up 3D scan and placing of the implant. To ensure the accuracy of navigation, patients were instructed to remain motionless, particularly the femur. After a satisfactory reduction of the fracture, the traction table, provides continuous and stable tractive strength for the lower extremity, preventing the femur moves or rotating. This ensured the reliability and accuracy of navigation. (iv)The equipment is relatively expensive and the operation table should meet the requirements of image collection. In addition, surgeons require education and training to operate the system, however, these are not available to all.

Nevertheless, there were several restrictions in this study: There was a potential risk of selection, confounding, and expertise bias due to the single-institution, retrospective study design. Considering that patients might not be able to accurately delineate some aspects of the Harris hip score system, such as deformity and range of motion, the pre-fracture HHS was not included in two groups in this study, this was one of the limitations in the present study. The greater final HHS in the study group may be related to the better pre-fracture functional status or the short follow-up time. In addition, the follow-up period was short and long-term complications such as osteonecrosis could not be predicted. Further studies using a larger sample size, extending the duration of follow-up, and recording the pre-fracture HHS are required to resolve this issue. Moreover, we only knew the number of fluoroscopic images between the two groups, and we did not measure the real ionizing radiation exposure for the patient and the operator. The use of dosimeters to determine individual exposure to ionizing radiation should be taken into account for future studies.

## Conclusions

FNS assisted by the orthopaedic surgery robot positioning system is an ideal method for the treatment of FNFs. This technique can effectively improve the efficiency of surgery and the success rate of one-time implant placement and reduces surgical trauma. Which in turn improved the prognosis. However, a stable anatomic reduction of the fracture and proficient skills of the robot navigation system is strongly warranted. In future research, we will expand the sample size and prolong the follow-up time to assess the occurrence of complications, such as osteonecrosis and nonunion.

## Data Availability

The datasets used or analyzed during the current study are available from the corresponding author on reasonable request.
